# Roughness-engineered potential wells for controllable ultrasonic cavitation: Multistage bubble capture–release mechanism revealed by multiscale experiments and simulations

**DOI:** 10.1016/j.ultsonch.2025.107610

**Published:** 2025-10-11

**Authors:** Yibo Suo, Xijing Zhu, Chenglong Bi, Linzheng Ye, Jing Li, Zuoxiu Li, Xiangmeng Li, Quan Cheng

**Affiliations:** a Shanxi Key Laboratory of Advanced Manufacturing Technology, North University of China, Taiyuan, Shanxi 030051, China; b School of Mechanical Engineering, North University of China, Taiyuan, Shanxi 030051, China; c School of Materials Science and Engineering, North University of China, Taiyuan, Shanxi 030051, China

**Keywords:** Ultrasonic Cavitation, Nanobubble, Molecular Dynamics

## Abstract

Controlling cavitation dynamics is essential for optimizing ultrasonic-assisted processing, targeted energy release, and damage mitigation. Here, we propose a structural strategy for tunable cavitation control using roughness-engineered surfaces that generate geometry-induced potential wells. Titanium alloy walls with varying porosity were fabricated via triply periodic minimal surface designs to systematically modulate roughness. High-speed imaging revealed that moderate roughness stabilizes bubble aggregation above the surface, whereas smooth walls fail to retain bubbles and excessive roughness induces perturbation-driven release. Specifically, in the 20 % porosity sample at t = 0.78 ms, multiple larger clusters formed at the center region of the wall, displaying asymmetric shapes and stretched edges, significantly increasing bubble retention time. Molecular dynamics simulations demonstrated that van der Waals–dominated short-range adsorption and localized low-energy zones extend bubble residence time, enabling stable capture. Excessive roughness, however, disrupts potential well uniformity, triggering asymmetric collapse and directed energy release. Integrating experimental and simulation results, we establish a multistage “capture–perturbation–collapse–release” framework for surface-induced cavitation control. This approach could potentially enable targeted cavitation control in ultrasonic cleaning, precision machining, and erosion prevention.

## 1 Introduction

Ultrasound is a non-invasive technology with broad applications [[1], [2], [3], [4]]. Ultrasonic cavitation refers to the process in which microscopic bubbles in a liquid form, grow, and collapse violently under an ultrasonic field [5]. This phenomenon is accompanied by extreme physical effects such as localized high temperature and pressure [6], strong shock waves [7], and microjets [8]. It has been widely applied in enhancing heat and mass transfer [8], surface treatment of materials [9], and minimally invasive medicine [10]. In various industrial processes such as metal processing [11], chemical catalysis [12], and cleaning [13], cavitation-induced impact and erosion on material surfaces [14] are particularly significant. It has been demonstrated that cavitation can effectively enable surface strengthening [15], void repair [16], and localized material removal [17], exhibiting great potential for microscale surface modification.

Most existing studies focus on the mechanisms of cavitation-induced effects on materials, such as erosion morphology evolution and degradation of fatigue performance. Current cavitation control methods typically involve tuning ultrasonic parameters (such as frequency [18], power [19], or exposure time [20]) or altering the liquid medium [21] to modulate overall intensity, thereby minimizing damage to sensitive materials. Although surface modifications for bubble control have gained attention, active strategies to control cavitation bubbles are still emerging. Previous studies, such as hydrophobic nanopatterned and laser-textured substrates, have explored bubble behavior control, but a comprehensive strategy remains under development. Our study addresses this gap by actively manipulating cavitation through surface roughness engineering, enhancing processing efficiency. The following table summarizes the key methods and focuses of current research, highlighting the significance of our work (Table 1.).

**Table 1:** Overview of surface engineering approaches for active cavitation control.

Method	Research focus	Key focus of our study
Nanopatterned coatings [22]	Investigates the behavior of nanobubbles on water nanostructured surfaces, focusing on the impact of porosity on bubble morphology, with X-ray scattering providing detailed bubble structure information.	Expands on roughness-induced cavitation control mechanisms, exploring bubble capture, stability, and release processes.
Wettability control surface [23]	Analyzes liquid wetting behavior through models, focusing on nucleation and growth of droplets on various surfaces, including Wenzel and Cassie–Baxter states.	Studies the effect of rough surfaces on bubble nucleation, using molecular dynamics simulations to analyze cavitation behavior.
Laser textured surface [24]	Investigates the effect of laser-textured surfaces on bubble sealing performance, using CFD simulations to analyze fluid dynamics and bubble behavior.	Focuses on cavitation nucleation mechanisms, combining experimental data with simulations to study bubble capture and release.

Surface roughness is a key factor influencing cavitation bubble behavior. Rough structures can modulate local pressure distribution, interfacial energy barriers, and surface tension conditions, thereby altering the nucleation sites, aggregation patterns, and collapse modes of bubbles. As a result, surface roughness can have a “capturing” or “releasing” effect on cavitation activity. Marin et al. showed that nanoscopic impurities [25] and interfacial defects [26] critically affect cavitation inception, providing important insights for controllable cavitation. If cavitation bubbles can be precisely manipulated through rational design of surface roughness, it would offer a novel approach for controllable cavitation applications. Recent studies confirm the decisive role of surface roughness in cavitation. Kadivar et al. showed that microscale roughness alters laser-induced bubble collapse, breaking symmetry and mitigating erosion [27]. Sarraf et al. emphasized in microfluidic cavitation that engineered defects govern nucleation and bubble evolution [28]. Building on these insights, our work advances to mesoscale roughness design, aiming to actively manipulate bubble capture and release via geometry-induced potential wells.

In this context, this study designs rough wall samples with varying porosity [29] using triply periodic minimal surface (TPMS) structures, fabricated via metal 3D printing. A standardized ultrasonic cavitation test platform is employed, and bubble behavior is visualized using high-speed imaging. To further elucidate the underlying mechanisms, a multiphase molecular dynamics model involving water, gas, and solid phases is developed. A two-dimensional sinusoidal surface is introduced to represent the rough wall, enabling the investigation of the dynamic response of bubbles near the structured surface.

This work aims to systematically reveal the capturing mechanisms of ultrasonic cavitation bubbles by rough surfaces through both experimental and theoretical approaches. It provides fundamental insights and technical guidance for controllable cavitation applications. The ultimate goal is to transform cavitation behavior control from passive modulation to active manipulation, and to explore structure-based precision cavitation strategies, offering theoretical and technological support for high-precision, low-damage ultrasonic-assisted processing.

## 2 Qualitative Experimental Analysis of Bubble Capture on Rough Surfaces

To investigate the influence of surface roughness on the behavior of ultrasonic cavitation bubbles, a series of controlled experiments were designed and conducted. Surface roughness was systematically modulated using triply periodic minimal surface (TPMS) structures, and samples with varying roughness levels were fabricated via metal 3D printing. A standardized ultrasonic cavitation platform was established, where excitation parameters were fixed, and a high-speed imaging system was employed to capture real-time bubble dynamics near the wall surfaces. A flat sample with 0 % porosity was used as the control group to enable direct comparison and evaluate the effect of roughness on bubble capture capability. The experimental observations provide critical references and validation data for subsequent molecular dynamics simulations.

### 2.1 Design and Fabrication of rough surfaces based on TPMS structures

To investigate the influence of surface roughness on the bubble capture capability during ultrasonic cavitation, and to enable both quantitative control of roughness and future engineering applicability, this study employs TPMS structures to design surfaces with varying roughness levels. TPMS structures are characterized by continuous curvature, high specific surface area, and tunable porosity [30], making them ideal for precisely engineering surface topographies with different roughness grades.

In this work, the Primitive-type TPMS was selected as the representative structure. Its implicit function is defined as [31]:

(1) $$\cos\left(\text{x}\right)+\cos\left(\text{y}\right)+\cos\left(\text{z}\right)=t$$

**Fig. 1:**
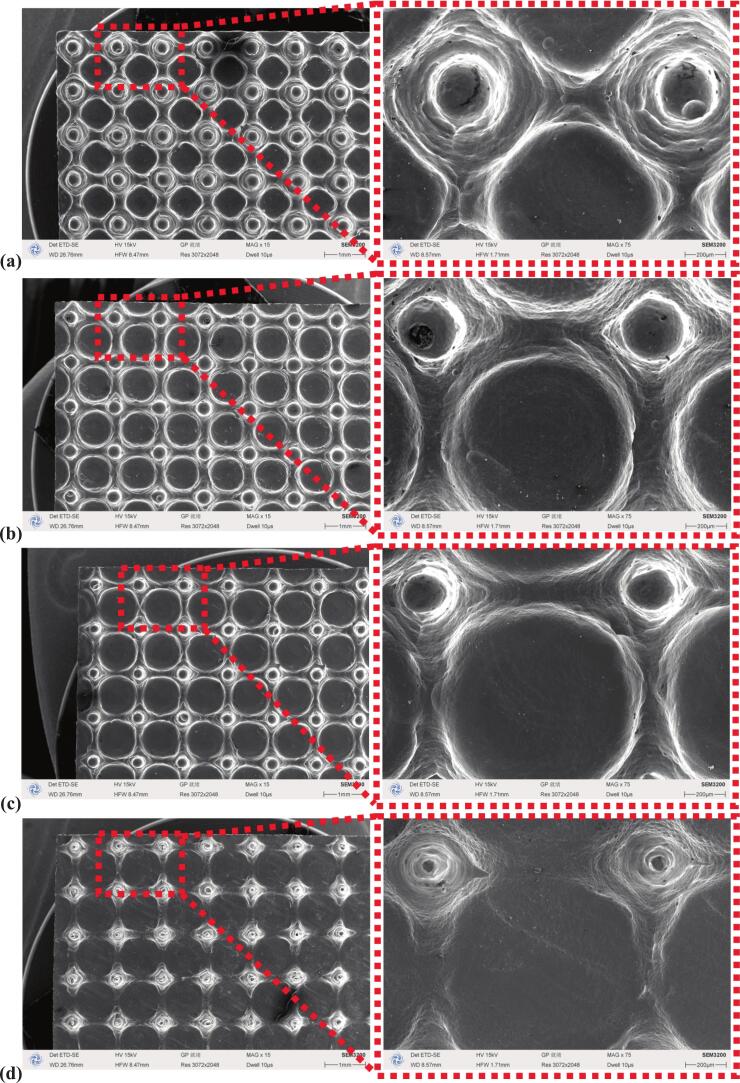
TPMS-Based Rough Wall Samples with Varying Porosities: (a) 5 %, (b) 10 %, (c) 15 %, and (d) 20 %.

The function parameter t in the TPMS implicit equation exhibits a strong linear relationship with the volume fraction and can be used to precisely control the porosity of the structure. By adjusting the t values, Primitive unit cell models with target porosities of 10 %, 20 %, 30 %, and 40 % were generated via Boolean operations in MATLAB and Materialise Magics. These models were subsequently imported into a selective laser melting (SLM) system to fabricate wall samples with porosities of 5 %, 10 %, 15 %, and 20 %, thereby representing surfaces with varying degrees of roughness. The fabricated samples are shown in Fig. 1. To provide a baseline for comparison, an additional group of flat wall samples with 0 % porosity was fabricated as a control group to validate the actual effect of surface roughness on bubble capture behavior.

To ensure high-quality surface interfaces, a standard surface treatment process was applied to all titanium alloy samples to remove oil residues and surface oxides. The cleaning procedure consisted of an alkaline treatment using a 10 wt% sodium hydroxide (NaOH) solution for 15 min to remove surface contaminants, followed by acid etching in a mixed solution of 8 wt% nitric acid (HNO_3_) and 3 wt% hydrofluoric acid (HF) for 5 min to eliminate the oxide layer.

### 2.2 Construction of High-Speed imaging observation platform and parameter Settings

A custom-built platform was developed to visualize cavitation bubble behavior. The ultrasonic system, provided by Hangzhou Chenggong Ultrasonic Equipment Co., Ltd., consists of a power supply, transducer, sonotrode, and a water tank. During the experiment, the ultrasonic frequency was set to 20  kHz with an output power of 640  W. The distance between the sonotrode tip and the sample surface was fixed at 10  mm. This setting was chosen to first generate cavitation bubbles in a stable acoustic field and then, under the assistance of acoustic streaming, deliver them toward the rough surface for controlled interaction. This spacing was precisely achieved using the micrometer scale of the sonotrode adjustment platform: the sonotrode was first brought into direct contact with the sample surface, and then retracted by rotating the adjustment knob counterclockwise until the 10 mm mark was reached. Deionized water was used as the working medium, and all experiments were conducted under ambient temperature conditions. The samples were mounted at the bottom of the water tank using a precision clamping fixture to ensure stable positioning. Special care was taken to align the sample center precisely with the focal region of the ultrasound field, thereby establishing a stable and controllable cavitation environment.

**Fig. 2:**
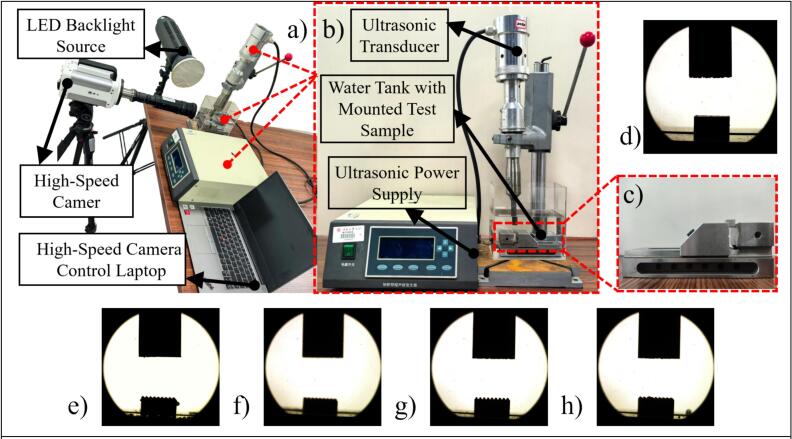
Ultrasonic cavitation experiment setup and pre-excitation cavitation region observation: (a) schematic of the high-speed imaging experimental platform, (b) system overview, (c) sample mounting structure, and (d–h) initial cavitation region images under different wall surface conditions.

Fig. 2(c) shows a close-up view of the sample mounting structure, while Fig. 2(d–h) present high-speed images of the bubble field under different porosity conditions before ultrasound excitation. These baseline images were used to eliminate background bubble interference and provide a reference for bubble dynamics after ultrasound application.

To capture the interaction between cavitation bubbles and the wall surface, a Photron FASTCAM SA-Z high-speed camera, coupled with a Photron high-speed digital microscopy system (FASTCAM Z16), was employed for real-time imaging. The frame rate was set to 6400 frames per second (fps), and an LED-150 T backlighting system was used to enhance image contrast and clarity, as shown in Fig. 2(a). The imaging system was focused on the region where bubble activity occurred in close proximity to the wall surface.

Under constant ultrasonic excitation, the high-speed imaging system continuously recorded the bubble evolution process. Each recording lasted 0.3 s, during which cavitation bubbles underwent dynamic capture and collapse near the rough surface. For each sample, three repeated recordings were performed to verify reproducibility, with the ultrasonic power switched on simultaneously with image acquisition. To highlight the key features during the bubble aggregation stage, one frame was extracted every five frames from the full video sequence, corresponding to a temporal resolution of approximately 0.78  ms. This approach generated a time-resolved image matrix that was used for analyzing the dynamic behavior of cavitation bubbles.

This experimental setup integrates structural rigidity, an open optical path, and high reproducibility in image acquisition, offering a robust and reliable foundation for studying the cavitation responses of rough surfaces with different structural parameters.

### 2.3 Time-Series Analysis of bubble evolution

**Fig. 3:**
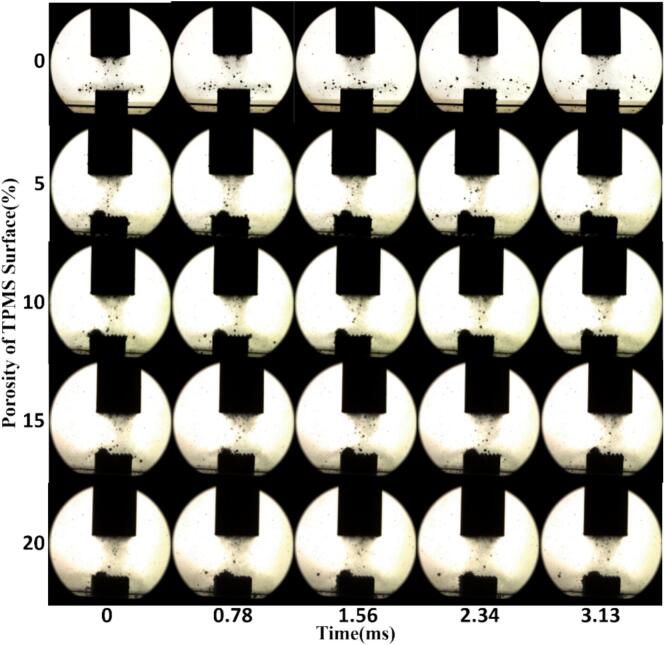
Time-sequenced evolution of cavitation bubble behavior near TPMS walls with different porosities.

The experimental results are shown in Fig. 3. From an overall perspective, bubble behavior is highly sensitive to surface roughness. As the porosity increases, cavitation bubbles exhibit a stronger tendency to accumulate and adhere near the wall surface, revealing a pronounced roughness-induced structural effect.

For the flat sample with 0 % porosity, no significant bubble accumulation was observed above the surface throughout the entire time series. After nucleation, bubbles rapidly floated upward or drifted laterally, showing highly dispersed trajectories and short lifetimes. No stable bubble retention occurred near the surface, indicating that the smooth wall lacks effective adsorption interfaces and potential well structures to support capture.

In contrast, the sample with 5 % porosity, representing a low-roughness surface, occasionally exhibited brief retention of small individual bubbles near the wall. However, the overall behavior remained dominated by upward floating and rapid dispersion. In the 10 % porosity sample, sparse and loosely distributed bubble clusters began to appear transiently above the surface, but failed to form continuous adhesion zones. When the porosity increased to 15 % and 20 %, the number of bubble clusters increased significantly. The clusters became more concentrated and persisted across multiple frames at consistent locations, demonstrating clear roughness-induced aggregation behavior. Notably, in the 20 % porosity sample, large and well-defined bubble clusters were observed directly above the wall. Some clusters even exhibited slight asymmetric deformation, suggesting that while capture capability was enhanced, surface-induced disturbances began to affect cluster stability.

More specifically, in the 15 % porosity sample at t = 1.56  ms, a well-defined bubble cluster formed symmetrically above the wall centerline. The cluster showed a complete boundary and fixed position, likely resulting from capture induced by a concave structural feature. In the 20 % porosity sample at t = 0.78  ms, multiple larger clusters formed at the center region of the wall, displaying asymmetric shapes and stretched edges. This further supports the hypothesis that high-roughness surfaces exert a dual effect of “capture and perturbation” on bubble clusters. In contrast, at the same time point, the 5 % porosity sample showed no visible bubbles near the wall, and only upward-moving bubble traces were captured. The control group exhibited no notable attachment throughout the time sequence, further confirming that surface roughness is a decisive factor in enhancing bubble capture capability.

In summary, rough surfaces significantly influence bubble behavior by altering local motion trajectories and adhesion locations. A clear evolutionary trend was observed—from non-aggregation to stable aggregation—as roughness increased, providing direct visual evidence of bubble capture at the macroscale. Although the experimental observations reveal the structural inducement capability of rough surfaces, they do not explain why bubbles preferentially attach to specific regions, nor do they quantify the underlying potential energy distribution or interfacial interaction forces. Therefore, it is necessary to introduce molecular dynamics simulations to explore the bubble–surface interaction mechanisms at the atomic scale and further elucidate the microscale origin of roughness-induced cavitation capture behavior.

## 3 Atomic-Scale Mechanisms of Bubble Behavior Regulated by Rough Surfaces

Building upon the macroscopic experimental observations, molecular dynamics simulations were conducted to further investigate the microscale mechanisms by which rough surfaces regulate cavitation bubble behavior. A multiphase water–gas–solid interfacial system was constructed, in which surface roughness was modeled using a two-dimensional sinusoidal profile. The dynamic evolution of bubbles under ultrasonic excitation was systematically simulated. This modeling approach provides insight into how varying surface roughness influences bubble capture capability, addressing the resolution limitations inherent in experimental techniques at the microscale. The results offer theoretical support and design guidance for structure-induced cavitation control strategies.

### 3.1 Rough surface modeling and Construction of the Gas–Solid–Liquid system

Given the transient nature of cavitation bubble formation, a pre-defined bubble was introduced in the simulation to represent the initial cavitation state. The simulation box was constructed with overall dimensions of 108.45 × 108.45 × 200 Å^3^ and consisted of three main components: a rough metallic surface, a liquid water region, and a bubble cavity. In addition, a rigid metal slab was placed at the top of the simulation domain to mimic the ultrasonic sonotrode, serving as both a boundary constraint and a source of external perturbation.

The cavitation bubble was initialized by creating a spherical cavity with a radius of 20 Å inside the liquid domain. The bubble center was positioned such that its distance from the rough sample surface was 40 Å, ensuring sufficient separation to avoid trivial overlap while still allowing strong interactions with the roughness features during subsequent oscillation and collapse.

The liquid and gas phases were positioned between the two solid walls, with a well-defined spatial arrangement and grouping strategy. This configuration provided a stable and physically meaningful foundation for analyzing the dynamic behavior of cavitation bubbles under the influence of surface roughness.

The rough surface was constructed based on the following two-dimensional sinusoidal function [32]:

(2) $$z=A\cdot \sin\left(\frac{2\pi x}{\lambda }\right)\cdot \sin\left(\frac{2\pi y}{\lambda }\right)$$

**Fig. 4:**
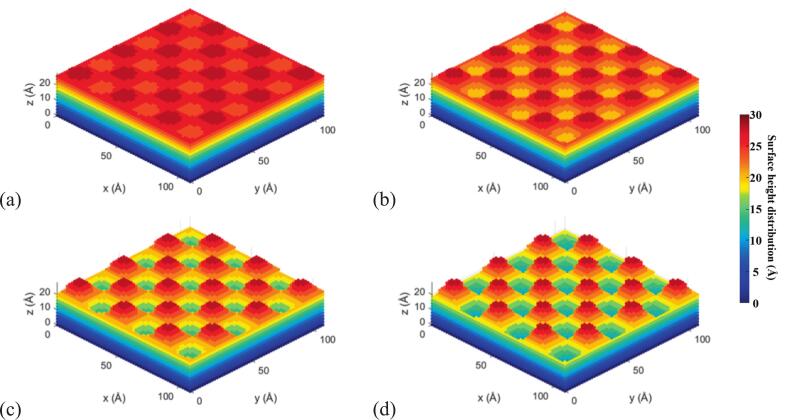
Theoretical 2D sinusoidal surface models with amplitudes of (a) 3 Å, (b) 5 Å, (c) 8 Å, and (d) 10 Å.

**Fig. 5:**
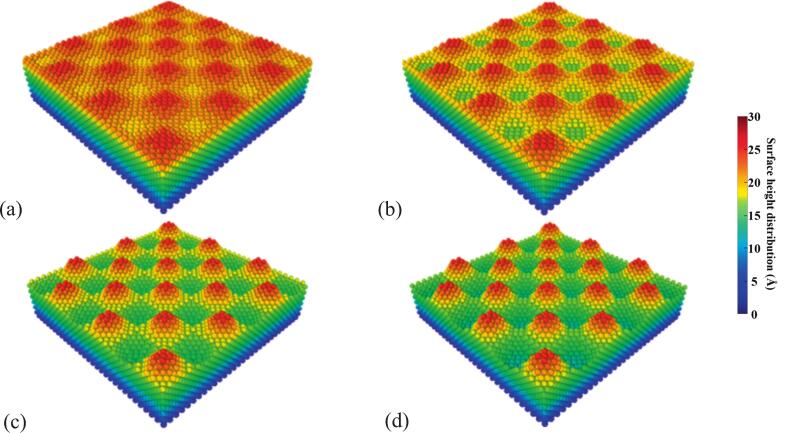
Simulated 2D sinusoidal surface models with amplitudes of (a) 3 Å, (b) 5 Å, (c) 8 Å, and (d) 10 Å.

In this equation, A represents the amplitude, which determines the roughness level of the surface, and λ denotes the wavelength, which was fixed at 36.15 Å. To simulate surfaces with varying degrees of roughness, five amplitude values were selected: A = 0, 3.0, 5.0, 8.0, and 10.0 Å, corresponding to different roughness intensities. The theoretical two-dimensional sinusoidal surface models and their corresponding atomic-scale simulation models are shown in Fig. 4, Fig. 5, respectively.

In our MD simulations, amplitudes of 3–10 Å were chosen because this range produces resolvable geometry-induced potential wells while avoiding artifacts from either thermal noise or unrealistic curvature. It should be noted that our aim is not to reproduce the absolute micron-scale roughness, but to reveal the underlying mechanisms at the nanoscale. Prior studies have shown that atomic steps and nanocavities often govern macroscopic phenomena such as cavitation [26], making the use of angstrom-scale roughness both reasonable and common practice.

### 3.2 Selection of potential Functions and parameter Settings

Simulations were carried out using the LAMMPS molecular dynamics package (Large-scale Atomic/Molecular Massively Parallel Simulator, version 17 Apr 2024) compiled with GNU C++ 9.4.0 and OpenMPI v4.0.3.

Due to the involvement of a complex multiphase particle system, a hybrid potential scheme was adopted to simulate the interactions between different particle types. The embedded atom method (EAM) potential, a widely used many-body potential for metallic systems, was employed to model interactions among metal substrate atoms. This potential accurately captures metallic bonding behavior, elastic properties, and surface characteristics [33].

In this study, the SPC/E three-point water model was employed, which has been widely used in cavitation and phase-transition studies and provides reasonable reproduction of liquid water properties. Although simplified three-point water models cannot fully reproduce all thermodynamic properties of liquid water, they significantly reduce computational cost while preserving molecular rigidity. This makes them well suited for large-scale liquid systems, especially in bubble-related simulations where local structural changes are not severe.

For non-bonded interactions—specifically between bubbles and water, and between bubbles and metal surfaces—a Lennard-Jones and Coulomb hybrid potential was used to capture van der Waals and electrostatic interactions [34]. This hybrid approach offers a simple formulation with widely available parameters and is particularly effective in modeling interfacial behavior at the molecular level, especially near the three-phase contact line involving gas, liquid, and solid phases.

Given the strong polarity of water molecules, electrostatic interactions between water molecules and between water and gas are non-negligible. The particle–particle particle–mesh (PPPM) algorithm was adopted to handle long-range electrostatics, providing a good balance between computational efficiency and accuracy, especially for large-scale systems involving directionally polar species. Long-range electrostatics were treated using the PPPM method with a real-space cutoff of 10.0 Å and a reciprocal-space accuracy of 10^−4^. This choice represents a standard compromise between accuracy and efficiency for water–solute–wall systems. Sensitivity checks with a tighter tolerance (10^−5^) and a larger cutoff (12 Å) confirmed that energies, forces, and bubble dynamics remained unchanged, verifying the reliability of the selected parameters.

### 3.3 Simulation procedure and Analysis of multiphase interfacial behavior

The simulation began with an energy minimization step to eliminate any unphysical atomic overlaps or high-energy local configurations that may exist in the initial structure. Performing energy minimization and initial equilibration prior to the production run is essential to ensure the accuracy and stability of subsequent bubble dynamics simulations [35]. This step effectively removes unfavorable atomic arrangements, such as those caused by randomly inserted water molecules or lattice distortions in the rough wall, which could otherwise lead to significant energy spikes during the early stages of the simulation, potentially resulting in system instability or distorted results.

After minimization, initial velocities were assigned separately to the water, bubble, and ultrasonic probe regions. The system was then equilibrated under a canonical ensemble (NVT) to reach thermal stability. Independent thermostatting was applied to different subsystems—including the liquid, gas, and solid phases—to enable sufficient exchange of kinetic and potential energy. This ensured that each region reached a physically reasonable thermal equilibrium state, which served as a reliable reference for the subsequent application of ultrasonic excitation.

Ultrasonic excitation was introduced by applying a periodic vibration to the rigid top metal slab, with an amplitude of 5 Å and a period of 10 ps. These parameters follow a scaling strategy [36] widely used in MD sonication studies, ensuring that the effective acoustic intensity corresponds to the experimental condition of 20 kHz, 640 W.

## 4 Multiscale Analysis of Roughness-Regulated Bubble Dynamics

### 4.1 Centroid Trajectory Analysis of cavitation Bubbles: Quantitative Evaluation of capture stability

**Fig. 6:**
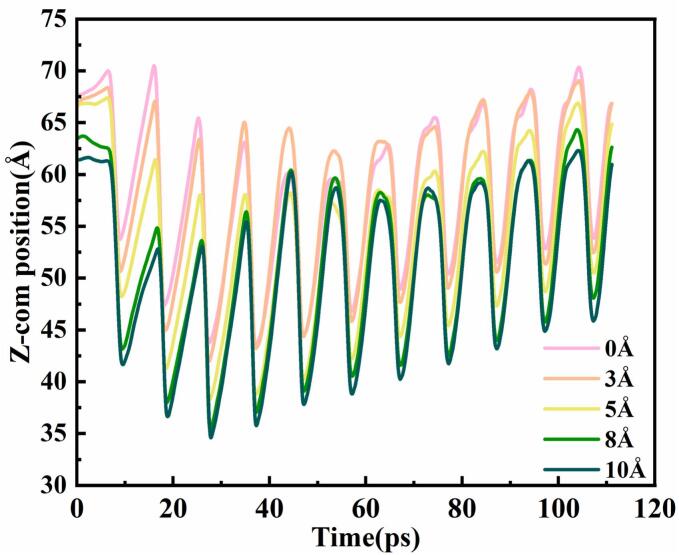
Temporal evolution of bubble Z-com position under different wall roughness conditions.

To quantitatively evaluate the influence of surface roughness on the spatial behavior of cavitation bubbles, the evolution of the bubble centroid position along the Z-axis was extracted over time, as shown in Fig. 6. The Z-axis position of the centroid reflects the bubble’s proximity to the solid wall and serves as a key indicator for assessing both capture capability and dynamic stability.

All roughness conditions exhibited a periodic oscillation trend in the Z-direction, indicating that the bubble undergoes typical expansion–contraction cycles under ultrasonic excitation and that the wall exerts an attractive effect in all cases. However, significant differences in centroid height were observed among the groups. On the smooth wall, the centroid remained at a relatively high position throughout the time series, suggesting that the bubble stayed far from the surface and was difficult to capture. When the roughness was set to 3 Å, the centroid position decreased slightly, indicating the onset of wall-induced attraction, though the motion remained unstable. At a roughness level of 5 Å, the centroid moved significantly closer to the wall and exhibited the smallest oscillation amplitude, indicating that the bubble was effectively captured and maintained the highest spatial stability. With further increases in roughness (e.g., 8 Å and 10 Å), although the centroid remained close to the surface, the oscillation amplitude increased markedly, suggesting that intensified surface-induced perturbations reduced capture stability.

In summary, moderately rough surfaces effectively induce bubble proximity and facilitate low-altitude oscillation near the wall, whereas overly smooth or excessively rough surfaces impair capture stability. A roughness level of 5 Å achieves the optimal balance between capture efficiency and dynamic stability.

This work aims to systematically reveal the capturing mechanisms of ultrasonic cavitation bubbles by rough surfaces through both experimental and theoretical approaches. It provides fundamental insights and technical guidance for controllable cavitation application

### 4.2 Analysis of Bubble–Wall interaction Energy: Adsorption strength and destabilization mechanism

**Fig. 7:**
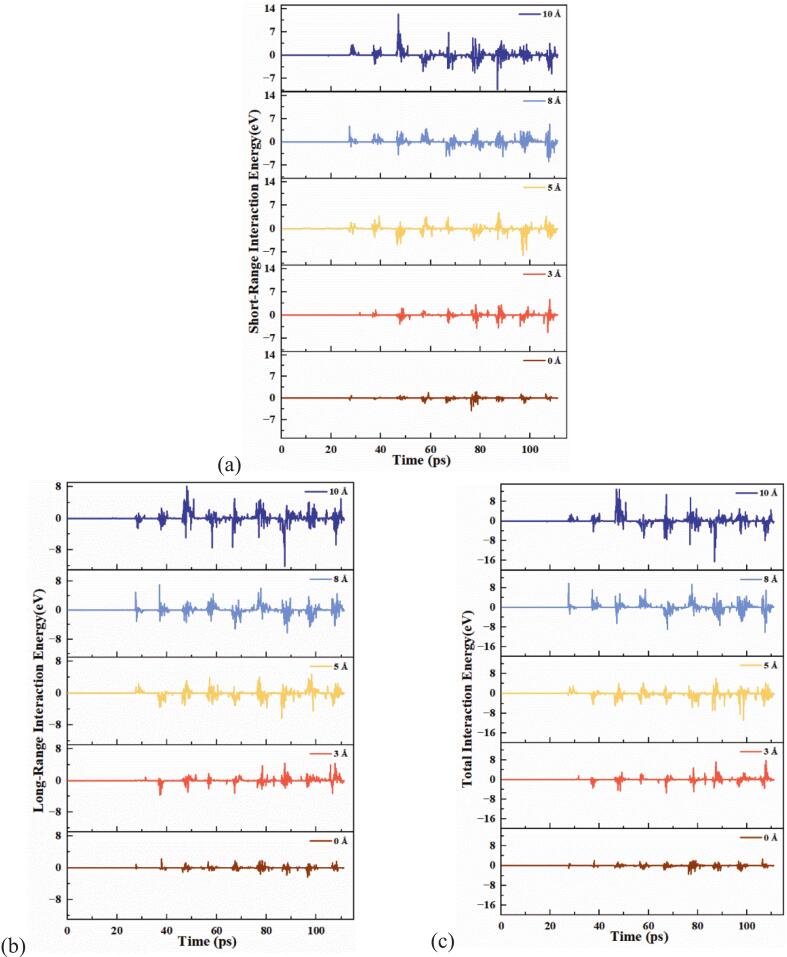
Time evolution of bubble–wall interaction energy under different roughness levels:(a) Lennard-Jones interaction energy, (b) Coulomb interaction energy, (c) Total interaction energy.

To investigate the influence of surface roughness on bubble capture capability, the time evolution of bubble–wall interaction energy was extracted under five roughness conditions (roughness levels: 0, 3, 5, 8, and 10 Å). In order to understand how surface morphology regulates bubble entrapment, stability, and dynamic response during cavitation, the interaction energy between the bubble and the wall was decomposed into short-range Lennard-Jones (L-J) potential, long-range Coulombic potential, and their total sum. The results are shown in Fig. 7.

Fig. 7a presents the time-resolved curves of the Lennard-Jones interaction energy under different roughness levels. As a short-range interaction, the L-J potential primarily reflects van der Waals attraction and repulsive forces, and thus serves as a direct indicator of physical adsorption between the bubble and the wall. The results show that the surface with roughness level 5 Å exhibits the most pronounced negative fluctuation in L-J potential throughout the ultrasonic cycle, along with a higher oscillation frequency. This indicates the strongest and most stable adsorption behavior at this roughness level., with the strong oscillations reflecting the significant capture ability due to the enhanced local interaction forces between the bubble and the rough surface. In contrast, both the smooth surface (roughness level: 0 Å) and the highly rough surface (roughness level: 10 Å) show significantly weaker L-J energy variations, suggesting either weak physical adsorption or frequent detachment events, indicating poor capture stability.

As shown in Fig. 7b, the variation in long-range interaction energy (Coulomb potential) is relatively small across all roughness conditions, and the fluctuation patterns do not change significantly with surface roughness. The Coulomb potential primarily reflects interfacial electrostatic interactions or polarity-induced structural rearrangements in the liquid phase, which are relatively limited in the present nonpolar wall–bubble system. Only under certain roughness levels, e.g., 3 Å and 8 Å, does the Coulomb contribution show a slight local enhancement. These results suggest that electrostatic interactions do not play a dominant role in bubble capture under the given conditions, but rather serve as a secondary modulation factor associated with local surface perturbations.

Fig. 7c presents the total interaction energy (Lennard-Jones + Coulomb) as a function of time. Overall, the surface with a roughness level of 5 Å again demonstrates the strongest interaction, characterized by sustained negative energy values and high-frequency fluctuations, indicating a prolonged and intense bubble–wall interaction. In contrast, for surfaces with roughness levels of 8 Å and 10 Å, although negative energy intervals still appear, the fluctuation patterns change significantly. These curves are marked by frequent positive–negative transitions and sharp oscillations, indicating that while high-roughness surfaces retain some capture capability, their intensified geometric perturbations disrupt stable adsorption, leading to bubble deviation, collapse, or rupture.

Combining the short-range and total interaction energy trends reveals that the Lennard-Jones component is the dominant physical contributor to bubble capture. Its temporal behavior closely matches that of the bubble centroid height. The roughness level of 5 Å represents an optimal configuration: it creates multiple stable adsorption potential wells without inducing excessive perturbation, thus achieving controlled capture and stable retention. In contrast, surfaces with insufficient roughness lack effective attractive fields, resulting in significant bubble drift. Excessively rough surfaces, while capable of attracting bubbles, introduce strong structural disturbances that destabilize the captured state and may trigger asymmetric collapse or localized rupture, often accompanied by microjets or shock wave emissions.

Notably, for the high-roughness surfaces (levels 8 Å and 10 Å), although the total interaction energy exhibits sharp fluctuations and frequent sign reversals—typically interpreted as signs of capture instability—the centroid trajectories and atomic potential maps suggest that these surfaces still possess short-term capture capability. However, due to their complex topography and high perturbation intensity, bubbles adhering to these surfaces are more likely to undergo asymmetric collapse. Such collapse can induce the release of microjets or pressure pulses, which in turn cause localized atomic rearrangements, manifested as abrupt transitions in the total interaction energy.

Therefore, high-roughness surfaces not only enable short-term bubble capture but may also promote controllable rupture behavior, making them potential candidates for directed cavitation energy release. This mechanism can be interpreted as a four-stage process: capture-perturbation-collapse-release, highlighting the role of rough surfaces not merely as passive stabilizers but as active controllers for cavitation energy manipulation.

### 4.3 Atomic potential energy distribution Analysis: Formation and evolution of Structure-Induced energy wells

**Fig. 8:**
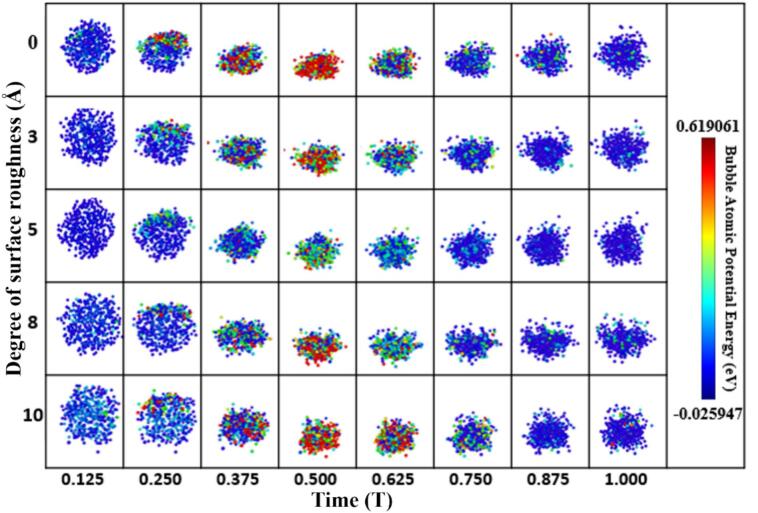
Atomic potential energy distribution of cavitation bubbles under different surface roughness levels.

To further elucidate the microscopic mechanisms by which surface roughness modulates bubble stability, the atomic potential energy distributions during bubble oscillation were extracted and compared under various roughness conditions, as shown in Fig. 8. The color-coded maps illustrate the spatial distribution of atomic potential energy, with blue representing low-energy regions and red indicating high-energy regions. These distributions serve to reveal both the internal energy state of the bubble and its correlation with the underlying wall structure, thereby enabling the identification of structure-induced potential wells.

Under the smooth wall condition (roughness level: 0 Å), the bubble exhibits a loosely organized structure with widespread regions of high atomic potential energy and poorly defined energy boundaries, indicating a mechanically unstable state prone to drift or deformation. Although no geometrically induced energy wells are present, weak bubble capture can still occur via short-range interactions such as van der Waals forces. However, these interactions lack sufficient confinement to maintain stable surface attachment.

As surface roughness increases, the internal potential energy of the bubble decreases significantly, and the energy distribution becomes more concentrated. At a roughness level of 5 Å, the bubble exhibits the most favorable characteristics: most atoms occupy low-energy states, and the energy field is highly uniform. This suggests a compact, energy-minimized configuration and signifies the highest degree of capture strength and structural stability. When the roughness increases further (levels 8 and 10 Å), although localized low-energy zones persist, numerous high-energy “hot spots” emerge, creating a non-uniform potential field. These fluctuations reflect enhanced wall-induced disturbances, leading to localized stress concentration and asymmetric deformations inside the bubble—phenomena that may trigger violent collapse and subsequent energy release.

The structural stability observed at moderate roughness is attributed to the formation of distinct potential wells induced by surface geometry. The periodic concave–convex features on the wall generate localized energy minima, into which bubble interface atoms are attracted when in proximity, thereby transitioning into a quasi-stable state. These structure-induced energy wells not only strengthen short-range adsorption forces but also significantly extend the bubble’s residence time near the wall and improve the symmetry of its oscillations. This mechanism accounts for the superior capture performance and stability observed at roughness level of 5 Å.

In contrast, smooth walls (roughness level of 0 Å) may enable brief adsorption, but due to the lack of structural confinement, their shallow energy gradients result in weak capture forces. Consequently, bubbles are prone to drifting or detachment under ultrasonic excitation. At a high roughness level of 10 Å, the wall topography becomes overly complex, leading to destabilized or transient energy wells and even interference zones, which undermine bubble stability and promote asymmetric rupture.

Importantly, high-roughness surfaces do not entirely lose their ability to capture bubbles. Analysis of both potential energy maps and centroid trajectories indicates that surfaces with roughness levels of 8 and 10 Å still exhibit phases of temporary bubble retention. However, the intensified interfacial perturbations from complex wall geometries lead to rapid destabilization post-capture, often resulting in asymmetric collapse and the emission of microjets or shock waves. This behavior can be described as a compound dynamic process: capture-perturbation-collapse-release. Rather than serving solely as stabilizers, high-roughness surfaces may function as active energy modulators, enabling targeted cavitation-induced damage or controlled energy release for applications such as localized surface processing and microstructural modification.

### 4.4 Integrated Analysis of Macroscale experiments and molecular dynamics simulations

At the macroscale, high-speed imaging clearly demonstrates that bubble clusters are more likely to form stable aggregation zones above moderately to highly rough surfaces—specifically, those with 15 % and 20 % porosity. In contrast, samples with 5 % or 10 % porosity, as well as the smooth control group, predominantly exhibit bubble trajectories characterized by dispersive motion or unstable surface attachment. These observations indicate that surface roughness can effectively regulate bubble behavior through geometric induction, enabling controlled cavitation bubble capture.

Correspondingly, molecular-scale simulations further reveal the underlying physical mechanisms governing bubble–wall interactions. As shown in Fig. 8, at a roughness amplitude of 5 Å, the Lennard-Jones (LJ) interaction energy exhibits the most pronounced negative fluctuations and the longest duration of energy minima, indicating that van der Waals–dominated short-range adsorption is strongest at this roughness level. In contrast, at 0 Å and 10 Å, the LJ energy curves display significantly dampened or frequently inverted fluctuations, suggesting that bubble attachment is unstable and prone to detachment or collapse. These results align well with the experimental observations—namely, the lack of clustering above smooth surfaces and the deformation of bubble clusters near highly rough surfaces.

Further insights are gained from the atomic potential energy distributions in Fig. 8. At moderate roughness, the wall structure induces well-defined potential wells that guide bubble atoms toward energetically favorable, symmetric configurations. The energy fields are concentrated and evenly distributed, reflecting a stable adsorption state. In contrast, highly rough surfaces generate localized stress concentrations and eruptive high-energy “hot spots,” indicative of intensified structural perturbations and unstable capture conditions. This energetic landscape interpretation provides a mechanistic explanation for the experimentally observed bubble shape rupture and instability.

This behavior can be described as a coupled multistage mechanism of “capture–perturbation–collapse–release.” In our macro experiments, we tested five groups of samples with porosities ranging from 0 to 20 %. The results showed a continuous increase in bubble capture ability from 0 to 15 % porosity. However, at 20 % porosity, a noticeable shift in bubble behavior occurred, indicating a transition from stable capture to collapse. This transition, characterized by bubble slippage and re-capture, suggests that while surface roughness and porosity promote aggregation, they also introduce perturbations that compromise overall stability. Similarly, in our MD simulations, we conducted tests with surface roughness values ranging from 0 to 10 Å. The centroid trajectories revealed that bubbles consistently moved closer to the wall and remained captured across the entire range. In the 0–5 Å roughness range, the interaction between the bubble and the surface increased, with a corresponding decrease in bubble potential, indicating stable adsorption. However, at 8 and 10 Å roughness, frequent positive–negative reversals in the interactions were observed, suggesting bubble collapse. The shockwaves and micro-jets generated during collapse caused transient interactions, resulting in an increase in potential energy. These findings highlight a clear shift in bubble behavior with increasing surface roughness, from stable capture to collapse-release. While the MD simulations pinpoint 5 Å roughness as optimal for bubble capture stability, the experimental results demonstrate that higher roughness and porosity enhance aggregation but also induce perturbations, diminishing the stability of the bubble clusters. The experimental and simulation results complement each other, providing a comprehensive understanding of how surface roughness and porosity influence bubble capture stability and illustrating the capture–perturbation–collapse–release mechanism within a specific range of surface conditions. It not only reveals the fundamental physical basis of bubble regulation via surface structure but also offers a theoretical foundation for structural design in applications requiring directional control of cavitation energy.

Overall, the multiscale findings demonstrate that van der Waals forces serve as the primary driving mechanism for bubble adsorption onto solid surfaces, while surface roughness modulates this behavior by introducing spatially localized energy minima: potential wells. These potential wells arise from the geometrical features of the surface and act as energy traps that amplify and stabilize the adsorption process. The synergy between physical attraction and geometrical confinement ultimately determines the capture state of cavitation bubbles at the solid–liquid interface.

## 5 Conclusions: Structure-Induced Cavitation Control Mechanisms

In this study, a series of surface models with varying degrees of roughness were constructed to investigate the cavitation bubble capture behavior and stability near solid walls under ultrasonic excitation. By integrating molecular dynamics simulations with visualization analysis, the regulatory mechanisms of surface roughness on cavitation dynamics were systematically explored. The main conclusions are as follows:

We demonstrate that moderate surface roughness creates uniform energy wells near the surface, optimizing bubble capture stability through enhanced van der Waals interactions. In contrast, smooth and excessively rough surfaces fail to maintain stable bubble attachment, with high roughness inducing perturbations that destabilize bubble retention.

A key finding of this work is that surface roughness not only regulates bubble capture but also facilitates asymmetric collapse, generating microjets and shockwaves. This collapse-release process is part of the capture–perturbation–collapse–release mechanism, opening new possibilities for directed energy release and targeted cavitation effects, which can be leveraged in applications such as materials processing and medical devices.

After temporary capture, bubbles near high-roughness surfaces often undergo asymmetric collapse due to severe interfacial disturbances, generating microjets and shock waves. This controllable rupture phenomenon suggests a promising route for directional energy release and localized cavitation-driven effects at the microscale.

In summary, surface roughness modulates cavitation bubble behavior through the synergistic regulation of van der Waals interactions and spatial energy field distributions. Moderate roughness achieves an optimal balance between capture strength and dynamic stability, providing both a theoretical foundation and design strategy for controlled cavitation applications. Meanwhile, the rupture-inducing characteristics of highly rough structures introduce a novel concept for targeted energy release, offering new avenues for practical engineering implementations.

The present study primarily emphasizes capturing representative qualitative features of cavitation bubble capture, aggregation, and collapse on rough surfaces, with mechanistic interpretation provided by molecular dynamics simulations. While this qualitative–mechanistic approach demonstrates cross-scale consistency, the absence of quantitative image-based metrics—such as bubble residence time, average cluster size, and deformation index—remains a limitation. Similarly, statistical treatments across multiple experiments (e.g., mean values and repetition-based analysis) have not been included in this work.

## 6 Statement of Originality

The authors declare that this manuscript is an original work, and the manuscript or part of it has not been published or submitted elsewhere.

## Ethical Approval

This study did not involve any human or animal subjects and therefore did not require ethical approval.

## CRediT authorship contribution statement

**Yibo Suo:** Writing – original draft, Visualization, Validation, Software, Methodology, Investigation, Formal analysis, Data curation, Conceptualization. **Xijing Zhu:** Writing – review & editing, Investigation, Formal analysis. **Chenglong Bi:** Writing – review & editing, Supervision, Resources, Project administration, Investigation, Formal analysis. **Linzheng Ye:** Writing – review & editing, Supervision, Software, Project administration, Methodology, Investigation, Formal analysis, Conceptualization. **Jing Li:** Writing – review & editing, Supervision, Software, Project administration. **Zuoxiu Li:** Writing – review & editing, Supervision, Software, Project administration. **Xiangmeng Li:** Writing – review & editing, Supervision, Software, Project administration. **Quan Cheng:** Writing – review & editing, Supervision, Software, Project administration.

## Declaration of competing interest

The authors declare that they have no known competing financial interests or personal relationships that could have appeared to influence the work reported in this paper.

## Data Availability

The data that support the findings of this study are available from the corresponding author upon reasonable request.
